# Mobile eye-tracking and neuroimaging technologies reveal teaching and learning on the move: bibliometric mapping and content analysis

**DOI:** 10.1093/psyrad/kkaf013

**Published:** 2025-05-23

**Authors:** Qi Li, Yafeng Pan

**Affiliations:** Department of Psychology and Behavioral Sciences, Zhejiang University, Hangzhou, 310058, China; Department of Psychology and Behavioral Sciences, Zhejiang University, Hangzhou, 310058, China; The State Key Lab of Brain-Machine Intelligence, Zhejiang University, Hangzhou, 310058, China

**Keywords:** bibliometric analysis, portable EEG, mobile fNIRS, mobile eye tracking, ecologically valid education

## Abstract

Mobile psychophysiological technologies, such as portable eye tracking, electroencephalography, and functional near-infrared spectroscopy, are advancing ecologically valid findings in cognitive and educational neuroscience research. Staying informed on the field's current status and main themes requires continuous updates. Here, we conducted a bibliometric and text-based content analysis on 135 articles from Web of Science, specifically parsing publication trends, identifying prolific journals, authors, institutions, and countries, along with influential articles, and visualizing the characteristics of cooperation among authors, institutions, and countries. Using a keyword co-occurrence analysis, five clusters of research trends were identified: (i) cognitive and emotional processes, intelligent education, and motor learning; (ii) professional vision and collaborative learning; (iii) face-to-face social learning and real classroom learning; (iv) cognitive load and spatial learning; and (v) virtual reality-based learning, child learning, and technology-assisted special education. These trends illustrate a consistent growth in the use of portable technologies in education over the past 20 years and an emerging shift towards “naturalistic” approaches, with keywords such as “face-to-face” and “real-world” gaining prominence. These observations underscore the need to further generalize the current research to real-world classroom settings and call for interdisciplinary collaboration between researchers and educators. Also, combining multimodal technologies and conducting longitudinal studies will be essential for a comprehensive understanding of teaching and learning processes.

## Introduction

Education is a crucial cornerstone of human wisdom and societal progress. Over the past two decades, researchers from various disciplines, including psychology and neuroscience, have increasingly focused on this field (Fischer *et al*., 2010; Feiler and Stabio, 2018). Accordingly, they have used psychophysiological tools to investigate educational topics. For instance, eye-tracking technology has been used to measure students' visual attention (Chuang and Liu, 2012), while electroencephalography (EEG) has been employed to assess cognitive load in multimedia learning settings (Antonenko and Niederhauser, 2010). Although these studies provide valuable insights into the cognitive and neural mechanisms underlying teaching and learning, their reliance on large, immobile equipment imposes limitations—particularly when studying interactive, dynamic, or movement-intensive educational contexts—by requiring participants to engage in tasks under strict movement constraints within controlled laboratory settings, which may differ from real-life learning conditions (Shamay-Tsoory and Mendelsohn, 2019; Davidesco *et al*., 2021; Stangl *et al*., 2023).

Notably, the development of mobile psychophysiological technologies, such as portable eye tracking, EEG, and functional near-infrared spectroscopy (fNIRS), has facilitated a paradigm shift. Despite existing challenges, such as increased susceptibility to motion artifacts and trade-offs between ecological and experimental control (Janssen *et al*., 2021), their inherent advantages—compact, battery-powered, light weight, and wearable—have enabled numerous researchers to investigate the neurocognitive mechanisms underlying ecologically valid teaching and learning (so-called “ecologically valid education,” Takeuchi *et al*., 2017; Bevilacqua *et al*., 2019; Coskun and Cagiltay, 2021). This is achieved by developing mobile technologies in real-world environments (e.g. classrooms) or in laboratory settings designed to closely simulate authentic educational activities (Janssen *et al*., 2021; Vigliocco *et al*., 2024). In this review, “ecologically valid education” refers to participants engaging in any teaching or learning processes involving knowledge, skills, or values in real-world or simulated settings, while experiencing minimal movement restrictions. However, a comprehensive investigation of the current research state, trends, and key topics in this field is still lacking. Moreover, understanding the impact of portable technologies on ecologically valid education remains insufficient.

Indeed, a few scholars have attempted to review the application of mobile technologies in educational research (e.g. Davidesco *et al*., 2021; Janssen *et al*., 2021; Gao *et al*., 2022). For instance, Dahlstrom-Hakki *et al*. (2019) discussed the potential of neurocognitive tools for addressing implicit learning in real classrooms and the associated challenges. Xu and Zhong (2018) conducted a systematic review of 22 articles on portable EEG technology in education, covering its applications, cognitive assessment aspects, and experimental procedures. While these studies have enriched our understanding of mobile tools in ecologically valid education, the reviews often focus on narrow perspectives (Li *et al*., 2019) and rely on qualitative methods, which may be subject to interpretation bias (MacCoun, 1998). Therefore, a comprehensive and quantitative analysis of the existing literature is needed for a more thorough understanding of the field.

To address the identified gaps, the current study conducted a bibliometric and content analysis to provide a comprehensive overview of research in the field over the past two decades (2004–2023). The year 2004 was selected as the starting point for the literature search as it corresponds to the earliest eligible publication in the Web of Science (WoS) database (i.e. Berka *et al*., 2004), which marked the inception of empirical research using mobile psychophysiological technologies in education-related contexts. Bibliometric analysis, a reliable method, involves the quantitative examination of extensive scientific data to map and interpret the cumulative knowledge and evolving trends within a field (Hood and Wilson, 2001; Donthu *et al*., 2021). This approach allows us to capture the current status and developing trends of the field, including annual article distribution, publication trends, influential works, prolific journals, authors, institutions, countries, and collaborative networks (Vošner *et al*., 2016; Song *et al*., 2019). Further, we employed keyword co-occurrence analysis, a specialized form of bibliometric analysis, to portray relationships among author keywords, followed by content mining to reveal major research themes and knowledge structures.

In summary, this study advances prior reviews (e.g. Xu and Zhong, 2018; Dahlstrom-Hakki *et al*., 2019) by integrating quantitative bibliometrics with qualitative content analysis. Unlike narrative reviews, these analyses offer a systematic and quantitative overview of the field's development. They allow researchers—especially those new to the area—to quickly grasp the research landscape, identify influential contributors, track emerging themes, and locate potential gaps. This, in turn, facilitates more informed research planning, collaboration, and dissemination strategies.

The remainder of this study is structured as follows. The Methods section outlines the data collection and analysis procedures. The Results section maps the bibliometric landscape of the field and examines the thematic content of five identified clusters. The Discussion section interprets the findings, highlights their implications, and proposes several future research directions.

## Methods

### Data retrieval and collection

In line with previous studies (Li *et al*., 2019; Yan *et al*., 2020), we selected the WoS as the sole database for analysis. Compared to other databases (e.g. Scopus), WoS is known for its rigorous scientific evaluation framework and high-quality list of peer-reviewed publications (Singh *et al*., 2021; Khan *et al*., 2022;). Moreover, different databases apply diverse counting rules for key metrics such as article citations, and combining them may lead to inconsistent or biased results (Schöbel *et al*., 2021). Following the search rules of the WoS database and informed by prior bibliometric research (Li *et al*., 2019; Yan *et al*., 2020), we formulated our search strategy to ensure adequate coverage of relevant studies while minimizing irrelevant records. A search was conducted in the WoS Core Collection using the query: (mobile OR portable OR wireless OR wearable) AND (fNIRS OR EEG OR eye-tracking) AND (teaching OR learning OR teach OR learn OR education OR educational) on 17 April 2024. The search was performed within the Science Citation Index Expanded (SCIE) and Social Sciences Citation Index (SSCI) with a time frame of 2004–2023 (inclusive). The initial search yielded 841 records. To ensure relevance and quality, we applied the following exclusion criteria: (i) publications not dated between 2004 and 2023, (ii) non-English language publications, and (iii) documents not categorized as articles or reviews. After this initial filtering, 786 publications remained. Next, two independent investigators conducted a manual screening of titles and abstracts. In cases where abstracts were unavailable or unclear, full texts were reviewed. Studies were included if they involved the use of mobile psychophysiological technologies—specifically mobile eye tracking, EEG, or fNIRS—in ecologically valid educational contexts, either in real-world settings (e.g. classrooms) or in laboratory environments simulating authentic educational activities. The inter-rater reliability between the two reviewers was 85%. Any disagreements were resolved through discussion until consensus was reached. This process resulted in 135 publications being retained for bibliometric analysis.

**Figure 1:**
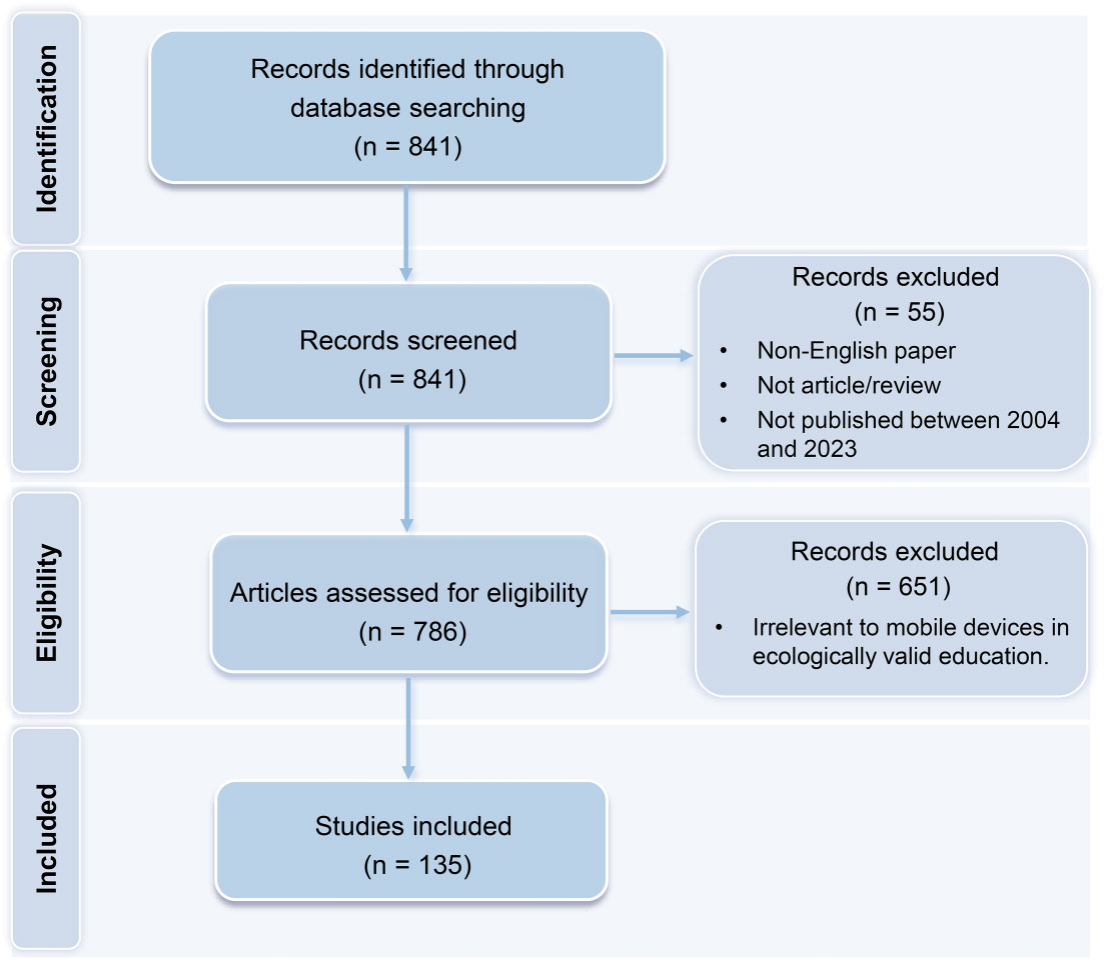
PRISMA flowchart for literature screening and selection.

Figure 1 presents the article screening and selection process, following the Preferred Reporting Items for Systematic Reviews and Meta-Analyses (PRISMA) guidelines (Page *et al*., 2021).

### Data analysis

#### Tools

Three tools were used for bibliometric analysis and network visualization: Microsoft Excel, VOSviewer, and Scimago Graphica. Microsoft Excel, with its built-in functions like filtering and sorting, allowed us to compile basic descriptive statistics such as the number of articles published annually and across various journals.

VOSviewer is a functional and user-friendly tool for creating and visualizing bibliometric networks based on scientific data (van Eck & Waltman, 2010) and is widely used in bibliometric studies (Arici *et al*., 2019; Achuthan *et al*., 2023). It can generate networks including scientific publications, authors, institutions, countries, or keywords, based on citation, co-authorship, or co-occurrence relations. Notably, compared with other commonly used tools such as CiteSpace and Bibliometrix, VOSviewer offers efficient visualization capabilities and employs clustering algorithms that are particularly effective for generating keyword co-occurrence and cluster networks with high interpretability, which facilitates subsequent content analysis (Li *et al*., 2019). Those terms belonging to diverse clusters in the networks are distinguished by different colors. In VOSviewer visualizations, items (e.g. keywords) are represented by both a label and a circle/frame, with their size reflecting the items’ weight (e.g. occurrences). Larger labels and the circles/frames indicate higher weights. These items can be interconnected through co-authorship, co-occurrence, or co-citation links, each with a strength that represents, for example, the number of publications co-authored by two institutions in the case of co-authorship links.

Data from VOSviewer were exported in GML format and imported into Scimago Graphica to generate collaborative networks (Hassan-Montero *et al*., 2022). Scimago Graphica allows the construction of complex visualizations by simple drag-and-drop interactions, following previous guidelines (Hassan-Montero *et al*., 2022; Zhang *et al*., 2023).

#### Bibliometric and content analysis

In the current study, bibliometric and content analysis were conducted. The analysis consisted of three steps. First, Microsoft Excel was used to calculate the annual number of articles and journal publications, with publishing trends visualized through bar charts. Second, co-authorship analysis was performed with VOSviewer, focusing on authors, institutions, and countries. Metrics such as publication counts, citations, and total link strengths were recorded to identify key contributors and their collaborations. Influential articles were determined through citation analysis in VOSviewer. Third, keyword co-occurrence analysis was conducted with VOSviewer to build networks showing keyword clusters. Each cluster's information was explored by reviewing the original articles linked to the keywords to gain insights into mobile technologies in ecologically valid education. The main research themes for each cluster were extracted and labeled, with synonymous terms, such as “functional near-infrared spectroscopy” and “fNIRS,” consolidated before analysis.

Metrics such as total publications (TP), total citations (TC), TC/TP, and total link strengths were recorded to identify key contributors and their collaborations. A higher TC/TP value indicates more citations per article on average; for example, authors with higher TC/TP values indicate that their papers receive more attention and citations in the academic community. A higher total link strength denotes a larger total number of publications co-authored by a given author/institution/country with others, suggesting that they are actively engaged in collaborative research.

## Results

### Publications and trends

**Figure 2:**
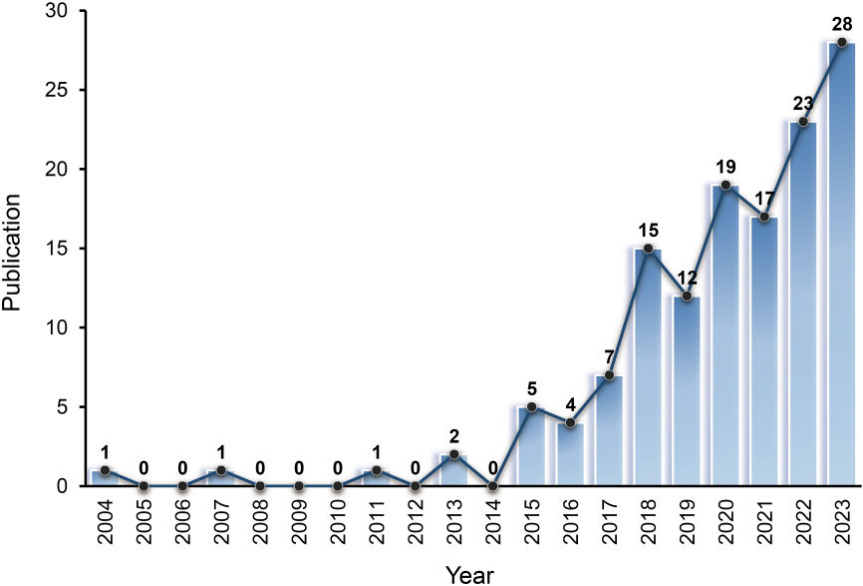
Annual publications between 2004 and 2023. Data were extracted from the Web of Science database on 17 April 2024, by conducting a search in the “topic” field using the following search string (mobile OR portable OR wireless OR wearable) AND (fNIRS OR EEG OR eye-tracking) AND (teaching OR learning OR teach OR learn OR education OR educational). The *x*-axis denotes publication year, and the *y*-axis shows the number of publications. Bar heights reflect annual publication counts, and the line indicates the overall trend over time.

Figure 2 shows the annual distribution of publications from 2004 to 2023. Between 2004 and 2014, the number of articles was low, with no more than two published per year, suggesting limited research interest in mobile technologies in ecologically valid education during that period. However, starting in 2014, there was a notable increase in publications, with annual outputs rising from an average of six articles per year from 2015 to 2017 to about 23 per year from 2021 to 2023, peaking at 28 in 2023. By the end of 2023, a total of 135 articles had been published. Despite some fluctuations in publication numbers (e.g. 2015–2016, 2018–2019, and 2020–2021), the overall trend has been steadily upward. This trend indicates that research on portable technologies in ecologically valid education has attracted growing scholarly attention (Fig. 2).

### Journals

Over the past two decades, a total of 97 peer-reviewed journals have published articles on this topic. Table 1 lists the journals with more than three published articles, with *Sensors* leading with nine publications. The remaining journals on the list each published three, collectively representing 22.22% of the total publication volume.

**Table 1:** Journals with the leading number of publications.

Journal	Publications	Citations	TC/TP	IF (2023)
*Sensors*	9	287	31.89	3.40
*Education and Information Technologies*	3	15	5.0	4.80
*Frontiers in Neuroscience*	3	179	59.67	3.20
*IEEE Transactions on Learning Technologies*	3	32	16.0	2.90
*Frontiers in Psychology*	3	65	21.67	2.60
*Frontiers in Human Neuroscience*	3	39	13.0	2.40
*Computers in Human Behavior*	3	151	50.33	9.0
*Journal of Visualized Experiments*	3	184	61.33	1.20

*Note*. TC = total citations, TP = total publications; IF = impact factor (Journal Citation Reports 2023).

As shown in Table 1, *Sensors, Frontiers in Neuroscience, Computers in Human Behavior*, and *Journal of Visualized Experiments* have each received over 150 citations. Notably, *Sensors* stands out with the highest citation count at 287. Although *Frontiers in Neuroscience, Computers in Human Behavior*, and *Journal of Visualized Experiments* have fewer total citations compared with *Sensors*, they each have a higher TC/TP (total citations/total publications) value, indicating that their articles are cited more extensively, with each article receiving over 50 citations on average.

Regarding the impact factor, *Computers in Human Behavior* leads with the highest rating of 9.0, though it has only published three relevant articles. The other journals vary in impact factor from 1.20 to 4.80, with an average of 2.93.

### Authors

A total of 569 authors have contributed to research in this field over the past 20 years. Table 2 presents the key contributors, with Kshitij Sharma leading with five published articles, ahead of other authors who each have three. Kshitij Sharma, Ido Davidesco, Suzanne Dikker, and Pierre Dillenbourg have all exceeded 100 citations. Notably, Ido Davidesco and Suzanne Dikker have the highest TC/TP value (62.33) and total link strength (28).

**Table 2:** Authors with the leading number of publications.

Author	Publications	Citations	TC/TP	Total link strength	Current affiliations
Sharma, Kshitij	5	125	25	17	Norwegian University of Science and Technology
Davidesco, Ido	3	187	62.33	28	Boston College
Dikker, Suzanne	3	187	62.33	28	New York University
Dillenbourg, Pierre	3	108	36	13	École Polytechnique Fédérale de Lausanne
Hannula, Markku S.	3	34	11.33	12	University of Helsinki
Müller, Katharina	3	77	25.67	11	Leibniz Universität Hannover

Among the most prolific authors, Kshitij Sharma focuses on optimizing intelligent educational systems through multimodal, data-driven approaches, such as the integration of visual gaze data (e.g. Sharma *et al*., 2020). Ido Davidesco conducts research in educational neuroscience, using psychophysiological techniques to uncover the cognitive and neural processes of teachers and students in classroom settings (e.g. Bevilacqua *et al*., 2019). Suzanne Dikker investigates the neurobiological foundations of dynamic social interaction and learning, with a particular emphasis on inter-brain synchrony (e.g. Dikker *et al*., 2020). Pierre Dillenbourg aims to design and apply human-computer interaction technologies to enhance learning and collaboration in authentic educational environments (e.g. Prieto *et al*., 2018). Markku S. Hannula explores learners’ affective experiences and cognitive processing patterns, particularly in the context of mathematics education (e.g. Salminen-Saari *et al*., 2021). Katharina Müller examines teachers' behavioral and cognitive characteristics—such as attention allocation—and investigates approaches to supporting teacher professional development (e.g. Goldberg *et al*., 2021).

**Figure 3:**
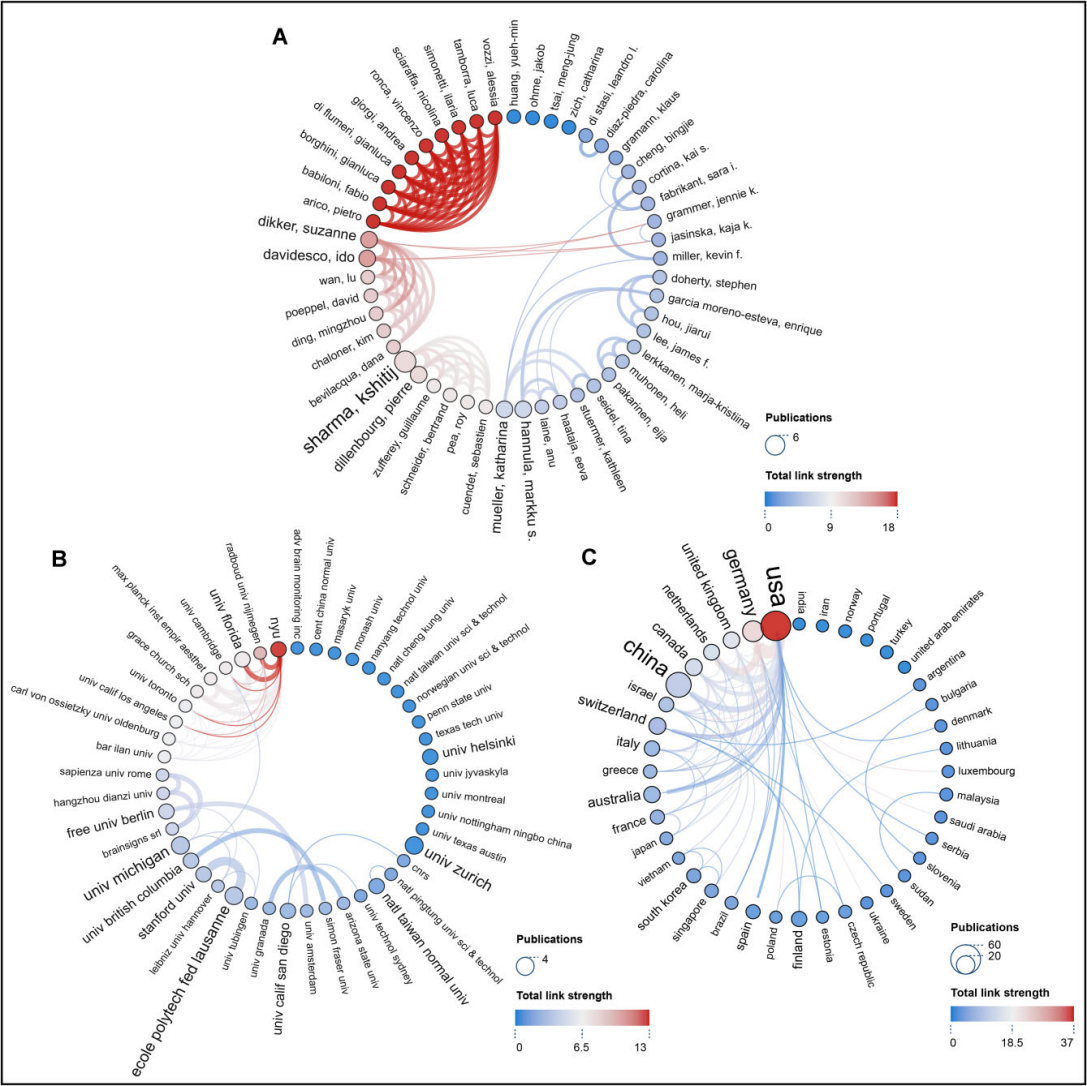
Collaborative networks. (**A**) Collaborative network of authors with more than two publications. (**B**) Collaborative network of institutions with more than two publications. (**C**) Collaborative network of countries. In the network, the size of the circles and labels denotes the number of an(a) author's/institution's/country's publications, which are positively correlated. The connecting lines indicate cooperative relationships, with thicker connecting lines representing more co-authored publications. The redder links and circles indicate stronger total link strength and more co-authored publications. The image was generated with Scimago Graphica software.

Figure 3A depicts the collaborative relationships among prolific contributors (publications ≥ 2, 49 authors). The size of the circles and labels represents the volume of an author's publications, with the larger size indicating more publications. The connecting lines denote cooperative relationships, with thicker connecting lines representing more co-authored publications. The redder links and circles indicate stronger total link strength and more co-authored publications. Figure 3A shows that most authors have collaborated with a stable and concentrated network of peers. For instance, Ido Davidesco, Dana Bevilacqua, and Suzanne Dikker are closely interconnected, forming a shared academic network. This group has worked together on projects such as using portable EEG hyperscanning (i.e. measuring multiple brains simultaneously) to study brain-to-brain synchrony and student–teacher dynamics in real classroom settings (Dikker *et al*., 2017; Bevilacqua *et al*., 2019).

### Institutions

From 2004 to 2023, a total of 268 institutions contributed to advancing this field. Table 3 highlights the most productive institutions (publications ≥ 3), with the University of Michigan, University of Zurich, and École Polytechnique Fédérale de Lausanne leading with four articles each, compared with other institutions that have each published three articles.

**Table 3:** Institutions with the leading number of publications.

Institution	Publications	Citations	TC/TP	Total link strength
University of Michigan	4	142	35.5	7
University of Zurich	4	124	31	4
École Polytechnique Fédérale de Lausanne	4	168	42	6
University of California San Diego	3	12	4	12
National Taiwan Normal University	3	23	7.67	2
University of British Columbia	3	25	8.33	7
Free University of Berlin	3	27	9	8
University of Helsinki	3	34	11.33	2
Stanford University	3	137	45.67	5
New York University	3	187	62.33	23
University of Florida	3	207	69	14

In terms of citations, the top three are University of Florida (207), New York University (187), and École Polytechnique Fédérale de Lausanne (168), followed by the University of Michigan (142), Stanford University (137), and University of Zurich (124). Notably, the University of Florida (69) and New York University (62.33) have significantly higher TC/TP values compared with other institutions, and their total link strength is also high.

Figure 3B illustrates the collaboration network among prolific institutions (publications ≥ 2, 45 institutions), showing that New York University engages with the highest number of collaborators. Conversely, some institutions, such as the University of Zurich and the University of Helsinki, have limited collaborative connections. Pearson correlation analysis was tentatively applied to explore the relationship between total link strength and TC/TP value, revealing an *r*-value of 0.56 (*P* = 0.07). This suggests that institutions with more extensive collaborations tend to produce articles with greater academic impact. This may be due to the resources and strength of influential institutions, which can lead to more impactful collaborative research.

### Countries

Researchers from 40 countries have contributed to this field over the past two decades. Table 4 presents the top 10 countries with the most publications (≥ 6) from 2004 to 2023. The USA, China, and Germany lead in terms of the number of both publications and citations, with the USA showing an exceptionally high citation count. China, as the only developing country on the list, has also made notable contributions. The USA also leads in TC/TP value. While Canada, Switzerland, and the Netherlands may not be prolific in publications, they have garnered a substantial number of citations. In terms of total link strength, the USA, Germany, Canada, the UK and the Netherlands are the top five, demonstrating a strong emphasis on international collaboration among their researchers.

**Table 4:** Countries with the leading number of publications.

Country	Publications	Citations	TC/TP	Total link strength
USA	44	1878	42.68	37
China	29	586	20.21	10
Germany	18	447	24.83	23
Canada	9	296	32.89	13
Switzerland	9	358	39.78	7
Australia	8	47	5.88	6
UK	8	83	10.38	15
Netherlands	7	224	32	14
Finland	6	71	11.83	2
Italy	6	40	6.67	6

Figure 3C illustrates the collaborative relationships among countries (publications ≥ 1, 40 countries), with the network of connections clearly showing the extensive and close exchanges between countries in advancing research in this field. The crisscrossed lines highlight the high level of international collaboration (Fig. 3C). However, some countries, such as India, Norway, and Turkey, could benefit from enhancing their cooperation and interaction with other countries to further their research contributions.

### Influential articles

Table 5 lists the top 15 most-cited articles from the selected time span. Among these highly cited articles, mobile EEG technology is the most prevalent subject, appearing in eight articles, including the top three. This indicates a strong focus on cognitive neural information related to teaching and learning facilitated by mobile EEG. Mobile eye-tracking technology is featured in five articles, while mobile fNIRS technology appears in only two articles. The lower representation of fNIRS may be due to its more recent introduction to the research community compared with EEG and eye-tracking technologies (Dahlstrom-Hakki *et al*., 2019).

**Table 5:** Articles with the leading number of citations.

Author (year)	Title	Journal	Citations	Mobile Technologies
Berka *et al*. (2007)	EEG correlates of task engagement and mental workload in vigilance, learning, and memory tasks	*Aviation, Space, and Environmental Medicine*	530	EEG
Berka *et al*. (2004)	Real-time analysis of EEG indexes of alertness, cognition, and memory acquired with a wireless EEG headset	*International Journal of Human-Computer Interaction*	258	EEG
Liu *et al*. (2013)	Recognizing the degree of human attention using EEG signals from mobile sensors	*Sensors*	218	EEG
Krigolson *et al*. (2017)	Choosing MUSE: validation of a low-cost, portable EEG system for ERP research	*Frontiers in Neuroscience*	171	EEG
Ayaz *et al*. (2011)	Using MazeSuite and fNIRS to study learning in spatial navigation	*Jove-Journal of Visualized Experiments*	166	fNIRS
Bevilacqua *et al*. (2019)	Brain-to-brain synchrony and learning outcomes vary by student–teacher dynamics: evidence from a real-world classroom EEG study	*Journal of Cognitive Neuroscience*	153	EEG
Xu and Zhong (2018)	Review on portable EEG technology in educational research	*Computers in Human Behavior*	102	EEG
Yang *et al*. (2018)	Examining creativity through a virtual reality support system	*ETR&D-Educational Technology Research and Development*	80	EEG
Cortina *et al*. (2015)	Where low and high inference data converge: validation of class assessment of mathematics instruction using mobile eye tracking with expert and novice teachers	*International Journal of Science and Mathematics Education*	79	Eye tracking
Harley *et al*. (2016)	Comparing virtual and location-based augmented reality mobile learning: emotions and learning outcomes	*ETR&D-Educational Technology Research and Development*	76	Eye tracking
Holper *et al*. (2013)	The teaching and the learning brain: a cortical hemodynamic marker of teacher–student interactions in the Socratic dialog	*International Journal of Educational Research*	66	fNIRS
Ackermann *et al*. (2015)	No associations between interindividual differences in sleep parameters and episodic memory consolidation	*Sleep*	66	EEG
Prieto *et al*. (2018)	Multimodal teaching analytics: automated extraction of orchestration graphs from wearable sensor data	*Journal of Computer Assisted Learning*	60	Eye tracking
Schneider *et al*. (2018)	Leveraging mobile eye-trackers to capture joint visual attention in co-located collaborative learning groups	*International Journal of Computer-Supported Collaborative Learning*	48	Eye tracking
Brügger *et al*. (2019)	How does navigation system behavior influence human behavior?	*Cognitive Research: Principles and Implications*	47	Eye tracking

These influential articles provide critical demonstrations of the use of mobile technologies in ecologically valid education. For instance, in EEG studies, Berka *et al*. (2007) examined EEG signals during cognitive tasks (e.g. image learning and memory tests) to evaluate the feasibility of wireless EEG for monitoring task engagement and mental workload, optimizing human–technology interaction. Bevilacqua *et al*. (2019) used EEG to investigate brain-to-brain synchrony among students and their teacher during biology lessons, exploring how this synchrony relates to learning outcomes and teacher–student dynamics (e.g. social closeness). In eye-tracking studies, Cortina *et al*. (2015) assessed differences in classroom monitoring abilities of expert and novice teachers by analyzing their visual gaze during mathematics instruction. Harley *et al*. (2016) used mobile eye tracking to track learners' visual gaze during history lessons with mobile augmented reality applications in both lab and outdoor environments. For fNIRS studies, Ayaz *et al*. (2011) used mobile fNIRS to monitor prefrontal cortex activity during spatial navigation learning. Holper *et al*. (2013) recorded brain activity between teachers and students during Socratic dialogues.

### Keyword co-occurrence analysis

**Figure 4:**
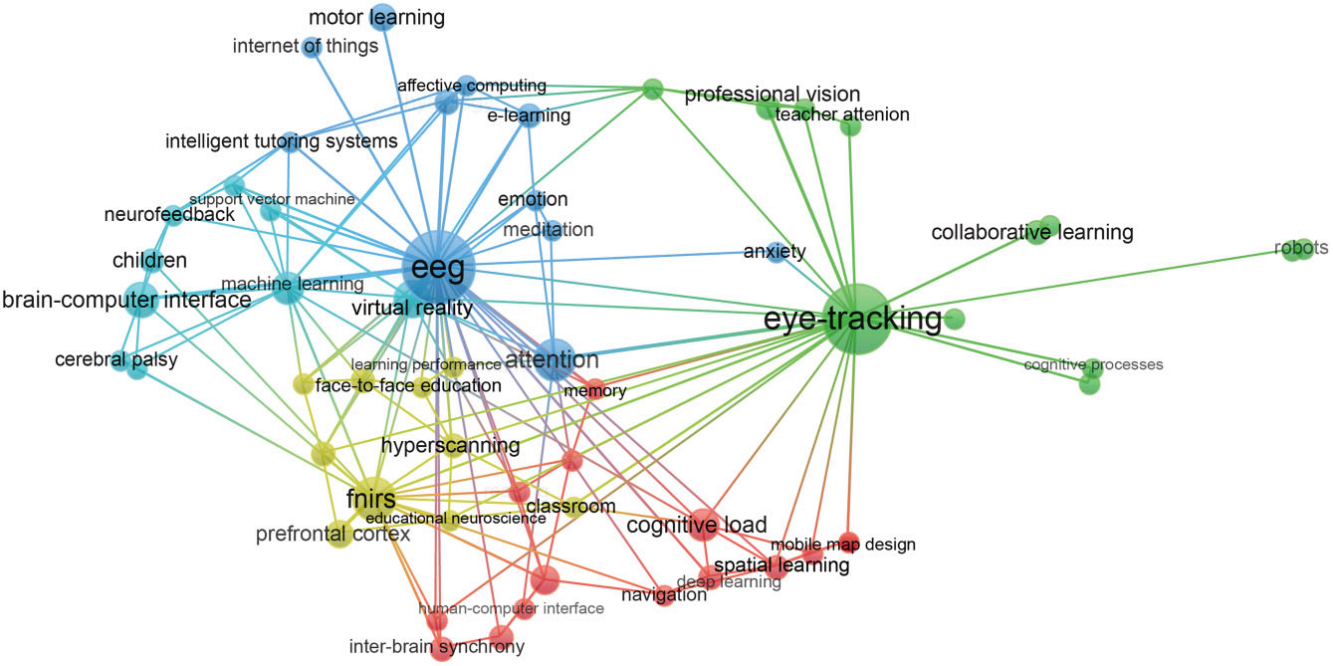
Keyword co-occurrence network (occurrences ≥ 2). Larger labels and circles indicate higher occurrences. Co-occurring keywords are connected by a link. The thicker the connecting line, the more co-occurrences between the keywords. Keywords belonging to different clusters are distinguished by color. The image was generated with VOSviewer software.

As illustrated in Fig. 4, we identified five major research clusters (i.e. the most prominent research streams in the field of education and mobile technologies) through keyword co-occurrence analysis, each distinguished by a different color and labels (each cluster label was generated by the researchers based on the terms included in each cluster):

1. Cluster 1: Cognitive and emotional processes, intelligent education, and motor learning (blue)
2. Cluster 2: Professional vision and collaborative learning (green)
3. Cluster 3: Face-to-face social learning and real classroom learning (yellow)
4. Cluster 4: Cognitive load and spatial learning (red)
5. Cluster 5: Virtual reality (VR)-based learning, child learning, and technology-assisted special education (cyan)

These clusters help us identify key research areas within the field, reveal the specific learning and teaching issues addressed by portable technologies, and illustrate how these technologies are (can, could be) contributing to ecologically valid education.

#### Cluster 1: Cognitive and emotional processes, intelligent education, and motor learning (blue)

Cluster 1 focuses on cognitive and emotional processes, intelligent education, and motor learning. Since portable EEG plays a key role in connecting these themes (Fig. 4), our content analysis primarily centers on studies involving it. EEG is a non-invasive neuroimaging tool with high temporal but low spatial resolution, detecting electrical field activity produced by pyramidal cortical neurons aligned parallel to the scalp (Lau-Zhu *et al*., 2019).

A key research focus of this cluster is on cognitive and emotional processes. It has been observed that emotional states strongly influence and interplay with various cognitive processes (e.g. memory, attention), thereby impacting behavior (Pessoa, 2008). Moreover, there is a reciprocal relationship between bodily experiences and emotional states, each affecting the other dynamically (Shamay-Tsoory and Mendelsohn, 2019). Therefore, cognitive and emotional processes are closely tied to bodily experiences in the context of teaching and learning. However, traditional neuroscience studies on cognitive and affective responses, constrained by large and immobile neuroimaging equipment, often limit participants’ movement during learning. This raises concerns about how well these findings represent learning conditions where participants can move freely. Currently, portable EEG is being used to address these critical issues. Mailhot *et al*. (2018) used wireless EEG to record participants’ brain signals without imposing strict physical constraints. The study examined their affective (e.g. anxiety, pleasure, and relaxation) and cognitive reactions (including attention, engagement, and interest) during e-health and e-learning interventions. Likewise, Balconi *et al*. (2023) used portable EEG in a real classroom setting to assess the effects of face-to-face versus remote learning on trainees and trainers. Their study evaluated cognitive (e.g. memory and attention), affective processes (e.g. emotional control), and social interactions during corporate training, with results showing that face-to-face learning was more effective.

Mobile EEG has also been instrumental in developing intelligent educational systems. Tailoring instruction to individual students’ learning experiences is crucial, yet teachers often struggle with the lack of standardized analysis methods, feedback systems, and observation techniques (Liu and Ardakani, 2022). Mobile technologies, however, are beginning to address these challenges. Studies involving portable EEG have focused on creating intelligent educational systems that enhance learning based on students’ natural learning experience. For example, Liu and Ardakani (2022) developed an e-learning system designed to personalize learning content based on students’ emotional states. Their system measures learners’ brainwaves using a portable EEG and processes the data with a machine learning algorithm to assess emotional states in real time during free-moving learning scenarios. The system then uses reinforcement learning to analyze and automatically recommend the most suitable learning materials to maintain a positive mood. Similar approaches were replicated in Alqahtani *et al*. (2020), which reported that mobile technologies could be effectively integrated into intelligent tutoring systems to support and enhance the learning process.

Investigating motor learning using portable EEG is another focus. Motor learning is vital for adapting to environmental changes and for applications in physical therapy and rehabilitation. Many studies on motor learning restricted participants’ movements (Krakauer *et al*., 2019; Haar and Faisal, 2020). Research in non-human animals suggests that neural activity patterns can differ between unrestricted and constrained movements (Nguyen *et al*., 2016; Svoboda and Li, 2018). Portable devices have transformed research on motor learning by enabling more naturalistic experiments without movement constraints. These devices allow for the investigation of neural activity in both laboratory and real-world environments. For example, Haar and Faisal (2020) used wireless EEG to study participants' post-movement beta rebound (PMBR) dynamics during a real-world motor learning task involving billiards. They discovered that participants exhibited varying PMBR dynamics—some showing increases and others decreases—suggesting that individuals might employ different learning mechanisms when faced with complex real-world tasks. Similarly, Filho *et al*. (2022) used portable EEG to monitor brain signals while participants played an open-skill video game. Their findings indicated that performance improvements from practice were associated with increased cortical activity, supporting the neural proficiency hypothesis. Based on these results, they proposed that neurofeedback interventions could enhance motor learning not only by helping performers manage brain activity but also by amplifying task-related brain networks.

#### Cluster 2: Professional vision and collaborative learning (green)

Cluster 2 is labeled as “professional vision and collaborative learning”. Fig. 4 highlights that the keywords in this cluster revolve around portable eye-tracking technology, which will be the focus of the following content analysis. Eye tracking, a useful psychophysiological tool, provides insights into cognitive processes by analyzing eye movements such as fixation count and saccade duration (Rayner, 1998; Lai *et al*., 2013).

A key focus in cluster 2 is using portable eye tracking to study teachers' visual attention and professional vision, which involves recognizing and interpreting salient classroom events based on professional knowledge (Seidel & Stürmer, 2014). For example, Haataja *et al*. (2019) employed mobile gaze tracking to explore how teachers' instructional intentions influenced their gaze behavior while guiding students through collaborative math problems in real classrooms. Their results indicated that the teachers' scaffolding intentions significantly affected their gaze patterns. Likewise, Pouta *et al*. (2021) used mobile eye tracking to compare the professional vision of student teachers and experienced teachers when teaching about rational numbers. They found that while both groups paid similar attention to mathematical concepts, their approaches to interpreting and guiding students' understanding of fractions differed.

Mobile eye tracking has also been used to study the joint visual attention of students during cooperative learning, where learners work together towards a common goal (Laal & Laal, 2012). For instance, Schneider *et al*. (2018) used mobile eye-tracking glasses to analyze student dyads’ joint visual attention while working on a warehouse layout. Their study revealed that higher levels of joint visual attention were associated with better collaboration quality. Salminen-Saari *et al*. (2021) explored students' joint attention during collaborative math problem solving in a real classroom. They identified phrases such as ‘verifying’, ‘watching and listening’, and ‘understanding’, noting that transitions between these phases often indicated successful interaction and enhanced collaboration. Others also used mobile eye tracking to study parent-infant interactive learning (Schroer & Yu, 2023) and informal learning scenarios such as museums (Mesmoudi *et al*., 2020) and outdoors (Kapaj *et al*., 2023).

#### Cluster 3: Face-to-face social learning and real classroom learning (yellow)

Cluster 3 is termed “face-to-face social learning and real classroom learning”. As depicted in Fig. 4, portable fNIRS (functional near-infrared spectroscopy) plays a core role in this cluster. fNIRS is a non-invasive neuroimaging technique that measures hemoglobin concentrations in the cerebral cortex (Lloyd-Fox *et al*., 2010; Scholkmann *et al*., 2014). Compared with EEG, fNIRS has a higher spatial resolution and is more tolerant to motion artifacts. This makes it increasingly popular across various research fields, including education.

A key focus of research in this area is social learning, which primarily occurs through interacting with others, such as teacher-student interaction (Tan *et al*., 2023). Historically, insights into the neural mechanisms behind social learning have mainly come from single-brain studies (Pierno *et al*., 2008; Tan *et al*., 2023). However, because social learning inherently involves dynamic interactions between at least two people, single-brain studies do not fully capture the neural complexities of social learning.

To fill this gap, researchers have used both immobile and portable fNIRS systems to investigate the neural mechanisms underlying face-to-face social learning between two naturally interacting individuals. In the present study, we focused specifically on portable fNIRS because of its higher ecological validity in naturalistic settings. Compared to immobile systems, portable fNIRS allows participants to engage in more natural bodily movements and interact in flexible environments (e.g. classrooms, Feng *et al*., 2025), which is particularly important for studying dynamic, real-world educational interactions (Davidesco *et al*., 2021). This is achieved using “hyperscanning”, a methodology that allows for the simultaneous measurement of brain activity from two or more individuals (Babiloni & Astolfi, 2014). A pioneering study employing wireless fNIRS-based hyperscanning recorded and analyzed prefrontal brain activity in teachers and students during Socratic dialogues (Holper *et al*., 2013). The study revealed a positive correlation in brain activity during successful educational interactions. In another study, the prefrontal cortex activity of teachers and students was measured during video game-based teaching and learning (Takeuchi *et al*., 2017). The findings showed that synchronized activity changes occurred in the left prefrontal cortex as the teaching–learning process progressed. These studies mark an important step into the era of two-person educational neuroscience, but they have primarily been conducted in laboratory settings, focusing on dyadic learning (Pan *et al*., 2022; Zhang *et al*., 2024). This raises questions about the extent to which these findings can be generalized to real-world classroom environments or larger group learning contexts (Davidesco *et al*., 2021; Feng *et al*., 2025).

In recent years, researchers have brought portable devices to real classrooms to examine the neural basis of collective teacher–student teaching, which is another research hotspot in this field. For example, Dikker *et al*. (2020) used mobile EEG hyperscanning techniques to record the brain signals of teachers and students during classroom teaching. They investigated the relationship between class time (early morning, mid-morning, and mid-afternoon) and students' alpha power—an indicator of attentional state (Klimesch *et al*., 2007)—as well as learning performance. Their findings revealed that students’ alpha power was lowest and quiz scores were highest during mid-morning classes, suggesting that this time may be optimal for learning and that class timing reflects students' brain states. Chen *et al*. (2023) used wearable EEG to monitor a group of high school students during Chinese and mathematics classes, exploring the neural mechanisms underlying successful learning in these disciplines. The study found that students with higher math exam scores exhibited stronger inter-brain coupling with all classmates, whereas those who excelled in Chinese had stronger inter-brain coupling with the top students in their class.

These studies highlight a noteworthy neurophysiological phenomenon: inter-brain synchrony, where the brain activity of interacting individuals shows temporal coordination. Inter-brain synchrony is emerging as a valuable neural marker for understanding social learning (Pan *et al*., 2018, 2022; Tan *et al*., 2023) and predicting learning performances (Zhang *et al*., 2024).

#### Cluster 4: Cognitive load and spatial learning (red)

Cluster 4 focuses on “cognitive load and spatial learning.” Cognitive load refers to the total cognitive resources engaged at any given time for processing information (Sweller, 1988; Baddeley, 2003). When the amount of information to be processed exceeds a learner's working memory capacity, cognitive overload occurs, which can lead to a plateau or even a decline in learning performance (Cheng *et al*., 2023). Therefore, precise measurement of cognitive load is essential for optimizing student learning and instructional design.

In a study involving medical education, researchers used portable EEG to assess the cognitive load of medical students and interns during simulated surgical training. They found that the EEG marker VC9 was highly sensitive to lower levels of cognitive load, and biomarkers such as delta, VC9, and theta (partially) decreased with reduced cognitive load and improved skill performance (Maimon *et al*., 2022). Additionally, beyond simply measuring individual cognitive load during natural learning or training (Whittier *et al*., 2020), there are ongoing efforts to develop methods for accurately monitoring and predicting cognitive load using signals from portable measurement devices (Yoo *et al*., 2023).

Another key research focus in this cluster is spatial learning. Navigation is a fundamental aspect of daily human life, involving the continuous processing and encoding of spatial information, such as routes, landmarks, and environmental layouts (Cheng *et al*., 2023). Cheng *et al*. (2023) used portable EEG to continuously monitor participants' brain activity while they navigated a virtual environment with the aid of a mobile map. They investigated how varying the number of landmarks displayed on the mobile maps influenced cognitive load during navigation. The study found that displaying five landmarks enhanced spatial learning without causing cognitive overload, compared with maps with three or seven landmarks. In another study, Kapaj *et al*. (2023) used a mobile eye-tracking device in outdoor settings to analyze participants’ gaze behavior during navigation tasks. They examined how different landmark visualization styles impacted spatial learning in wayfinding experts. The results indicated that while experts’ spatial learning improved when they focused their visual attention on the environment, the style of landmark visualization did not affect the direction of their attention between the map and the surroundings.

#### Cluster 5: VR-based learning, child learning, and technology-assisted special education (cyan)

Cluster 5 is labeled as “VR-based learning, child learning, and technology-assisted special education.” VR, a computer simulation system, can create immersive virtual worlds by generating interactive experiences involving vision, hearing, and touch (Yang *et al*., 2019) and has become widely used in educational settings. Research has employed mobile devices to investigate the neural processes of learners engaged in VR-based educational activities. For instance, Wen *et al*. (2023) equipped participants with a VR head-mounted display and had them use a controller in a VR environment to perform a virtual Morris Water Maze task aimed at training spatial cognition while using a portable EEG device to record their brain activity. The study found that VR-based spatial cognitive training effectively improved participants’ spatial abilities and influenced their neural activity patterns. Additionally, other research has explored how VR affects cognitive processes such as flow, attention, and meditation, using wearable EEG devices (Yang *et al*., 2018; Sumardani and Lin, 2023), and developed evaluation models for assessing students’ immersive experiences during VR learning using support vector machine and wearable recordings (Guo *et al*., 2022).

In cluster 5, a major research theme involves using portable fNIRS to examine children’s brain activity during natural learning and training. For example, in a clinical study, Perpetuini *et al*. (2022) used portable fNIRS to investigate the neurophysiological processes in children with cerebral palsy undergoing robot-assisted gait training (RAGT). They found that fNIRS effectively detected changes in brain activity associated with RAGT, which correlated with improvements in motor function. Additionally, Jasińska and Guei (2018) employed portable fNIRS in rural sub-Saharan Africa to measure brain activity in primary school children engaged in cognitive tasks such as reading. Their work led to the development of a field neuroimaging protocol aimed at better understanding reading development in environments at high risk for illiteracy.

Another research focus is technology-assisted special education, particularly through the use of brain–computer interfaces (BCIs). For instance, Kandemir and Kose (2022) used a lightweight wearable EEG headset to track brain signals from deaf children during physical activity-based games. They designed human–computer interaction games intended to enhance the children’s attention, thereby improving their learning efficiency. Additionally, Antle *et al*. (2018) developed a mobile neurofeedback BCI system with an EEG headset to measure the brain activity of illiterate children directly in their school environment. This system provided real-time neurofeedback during learning games and was found to help children from impoverished backgrounds develop self-regulation skills for managing anxiety and attention.

**Figure 5:**
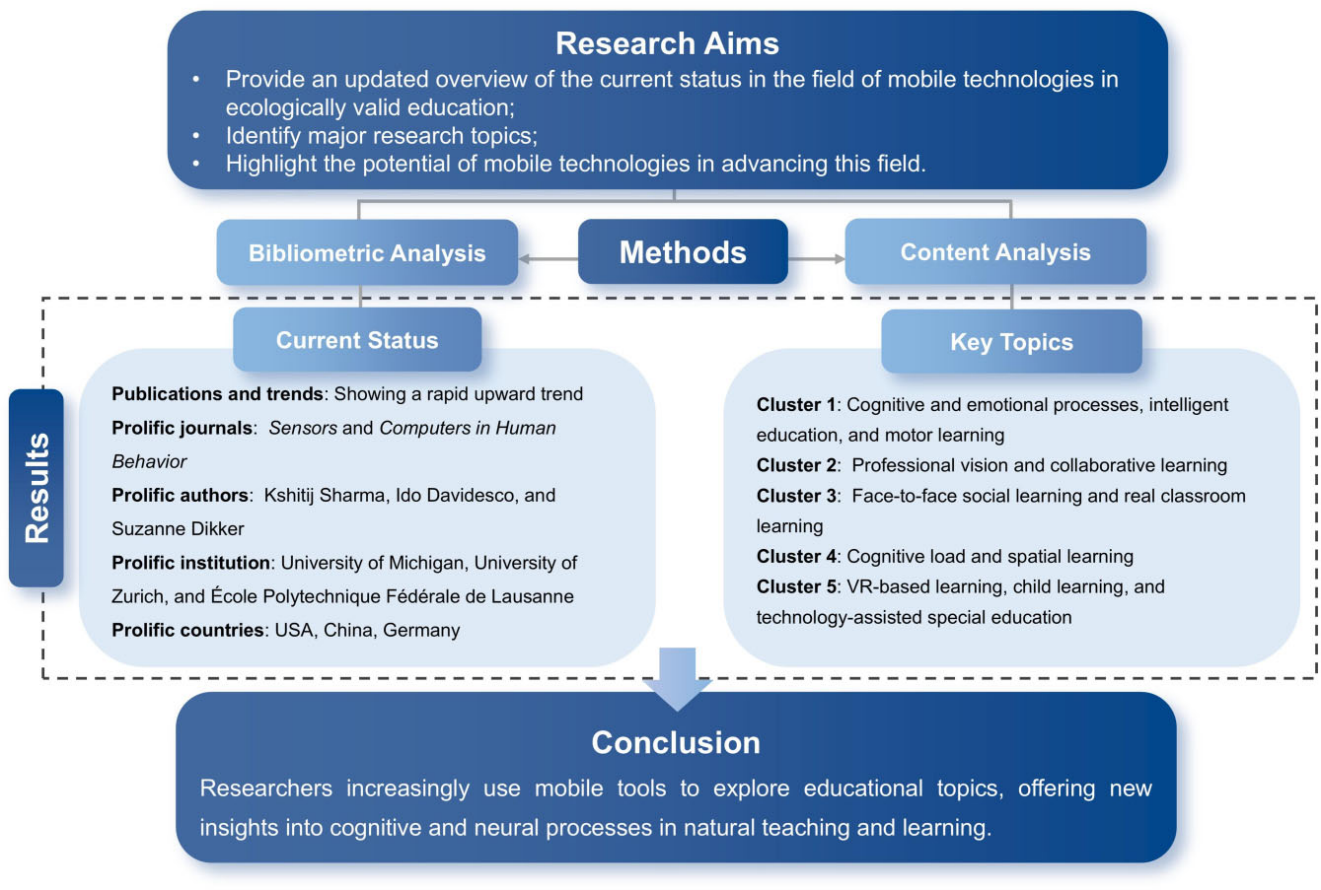
Overview diagram of the current study.

To sum up, we created an overview diagram to encapsulate the key findings of this study (see Fig. 5). Centered on the study’s motivation, we performed a bibliometric and content analysis to reveal the current state of research and the main themes in the field, emphasizing the distinctive role of mobile technologies in promoting ecologically valid educational research. Mobile tools have initiated a new era of research into natural teaching and learning, enhancing our understanding of education.

## Discussion

The current study conducted a bibliometric and content analysis of articles published over the past two decades on the use of mobile technologies in ecological validity education. The results demonstrate that this field is moving forward rapidly. The identified themes in five clusters highlight the diverse applications and potential of mobile technologies in educational research. The details are further elaborated below.

First, the bibliometric analysis results indicate a significant rise in the number of publications from 2004 to 2023. This increase may be linked to advancements in technology and a greater emphasis on ecological validity (Brockington *et al*., 2018; Shamay-Tsoory and Mendelsohn, 2019; Stangl *et al*., 2023). Among the leading journals, *Sensors* stands out as the most prolific in terms of publications and citations. However, its total citations per paper (TC/TP) and impact factor (IF) are lower compared with those of *Computers in Human Behavior*.

Our analysis also highlights Kshitij Sharma, Ido Davidesco, and Suzanne Dikker as the leading authors in terms of both publication and citation counts, as well as their prominence in collaborative networks. Researchers may benefit from following their work closely or seeking collaboration with them to gain useful insights into the field. In addition, the University of Michigan, University of Zurich, and École Polytechnique Fédérale de Lausanne are identified as the most prolific institutions in this area. Meanwhile, the field has attracted attention from researchers across 40 countries, with the USA, China, and Germany being significant contributors. This high level of international collaboration can be attributed to factors such as economic development, policy support (Hou *et al*., 2021; Matveeva *et al*., 2022), and pre-existing academic relationships, such as those between supervisors and former graduate students within the same research group.

Among the top 15 influential articles, there is a notable presence of studies using mobile EEG (e.g. Berka *et al*., 2007; Krigolson *et al*., 2017). These are followed by studies using mobile eye tracking (e.g. Cortina *et al*., 2015; Prieto *et al*., 2018) and fNIRS (e.g. Ayaz *et al*., 2011; Holper *et al*., 2013). Researchers interested in these technologies can refer to these articles for valuable insights into applying them in ecologically valid educational settings.

To our knowledge, few studies have conducted bibliometric analyses specifically focusing on the application of mobile technologies in ecologically valid educational research. This study aims to fill that gap by providing a quantitative overview of the current research landscape. By extending previous qualitative reviews (e.g. Dahlstrom-Hakki *et al*., 2019; Davidesco *et al*., 2021), our analyses offer complementary insights that can serve as valuable references for future investigations in this field.

Second, the keyword co-occurrence analysis identified five distinct clusters, which were then used to extract research themes through content analysis. Specifically, *Cluster 1* reveals that mobile EEG has been employed to reveal the cognitive and emotional processes in individuals without strict physical constraints during learning activities, such as e-learning. Furthermore, it has been leveraged to develop intelligent educational systems and to explore neural mechanisms underlying motor learning in real-world settings. Studies within *Cluster 2* utilize portable eye tracking to investigate teachers’ professional vision in authentic classrooms and to study joint visual attention among students during collaborative learning. *Cluster 3* highlights the critical role of portable devices in uncovering the neural foundations underlying face-to-face dyadic learning and real classroom learning. *Cluster 4* shows that portable tools enable assessment of cognitive load and exploration of neurocognitive processes during spatial navigation learning. *Cluster 5* demonstrates the potential of mobile devices in examining the brain bases during VR-based learning. It also underscores their key role in unveiling brain activity when children learn and developing BCI systems for special populations. Taken together, our findings suggest that mobile technologies are reshaping educational research across several dimensions: they enable the expansion of study settings from controlled laboratories to naturalistic environments, facilitate the inclusion of more diverse participant groups—including children and special populations—and foster a shift from individual-focused research to multi-person, interaction-based approaches. These developments can guide researchers in selecting meaningful research questions and designing more ecologically valid studies.

The findings also have practical implications for policy-makers and educators. Policy-makers might consider launching pilot programs in which professional researchers and teachers use mobile devices in classrooms to measure students’ cognitive and neural states in real time, thereby enhancing interaction and teaching quality. At the same time, such initiatives must be accompanied by clear data governance policies and robust ethical frameworks to protect individual privacy (Davidesco *et al*., 2021). Educators, on the other hand, can employ mobile devices (e.g. portable eye-trackers) to reflect on their own teaching behaviors and inform professional development.

While the field of mobile technologies in ecologically valid education is expanding rapidly, there are several areas that warrant further consideration. First, there is still a considerable distance to achieving generalization of current findings to real-world settings. This field is not fully developed, with most experiments taking place in the controlled laboratory. Given the complexity of real-world conditions, the applicability of these lab-based findings to everyday situations requires further validation (Stangl *et al*., 2023). It is important to recognize that data collection in dynamic real-world environments inevitably introduces more noise, highlighting the need for the development of new analytical tools and methods to improve signal-to-noise ratios. Moreover, while mobile devices offer reduced control over participants compared with traditional methods, their relatively large size can impact participants’ comfort and naturalness during learning, potentially influencing the results. Recent advances have introduced smaller and more transparent wireless devices, such as ear EEG (Kaveh *et al*., 2024), which could serve as promising tools.

Second, it is essential for researchers from interdisciplinary fields, such as psychology and neuroscience, to strengthen and maintain collaboration and communication with frontline educators. On the one hand, teachers can provide researchers with first-hand information about real-world education, for example, which students enjoy interacting with teachers, and what characteristics these students have. Teachers can also act as a “touchstone” to validate findings from ecologically valid education studies and in turn provide feedback to advance further study. On the other hand, researchers can not only facilitate educators’ understanding of their students by sharing professional knowledge (e.g. cognitive development, learning motivation, and brain plasticity) with them but also provide scientific suggestions for teachers’ educational practices.

Third, most current educational research using mobile devices remains cross-sectional. While these studies have brought us valuable insights, there is a need to fully use portable devices by incorporating longitudinal studies. Longitudinal designs enable researchers to uncover the trajectories of cognitive neural processes underlying teaching and learning and examine the bidirectional and cross-lagged relationships between these processes and educational factors (Alonso *et al*., 2024). This approach also allows us to identify the factors that do not show immediate and significant effects but may have a substantial impact over time through the accumulation of effects. Consequently, educational researchers can obtain findings that have important theoretical and practical implications.

Fourth, current studies on ecologically valid education predominantly rely on single-modality neurocognitive devices. Each of these devices has their own merits: mobile eye tracking can provide information about visual attention and processing, EEG unravels the temporal dynamics of cognitive processes, and fNIRS monitors the changes in the brain’s activity during specific tasks (Dahlstrom-Hakki *et al*., 2019). Future studies could benefit from integrating multimodal portable devices (Baceviciute *et al*., 2022; Sui *et al*., 2023).

Finally, several important research gaps remain. For example, in the context of global aging, there is an urgent need to explore how mobile technologies can support cognitive training and digital learning among older adults, a population well-suited for such interventions due to the flexibility of mobile devices. Additionally, the rapid integration of generative artificial intelligence (AI), such as ChatGPT, into education is attracting wide attention (Polat *et al*., 2024). Future studies could examine the long-term neural and cognitive impacts of AI-assisted learning using portable technologies. Researchers might also leverage cognitive–neural data to inform the design of adaptive AI learning systems that respond to learners’ real-time needs.

The current bibliometric review has several limitations. First, it focuses exclusively on peer-reviewed literature in English from the WoS database. Expanding the analysis to include other well-established databases such as Scopus and PubMed, as well as incorporating additional languages and document types (e.g. conference proceedings), may provide a more complete picture of the field. Second, the absence of author keywords in some articles may introduce bias into the keyword co-occurrence analysis. Lastly, the interpretation of keyword clustering maps is qualitative and inherently subjective.

## References

[bib1] Achuthan K, Nair VK, Kowalski R et al. (2023) Cyberbullying research—Alignment to sustainable development and impact of COVID-19: bibliometrics and science mapping analysis. Comput Hum Behav. 140:107566.

[bib2] Ackermann S, Hartmann F, Papassotiropoulos A et al. (2015) No associations between interindividual differences in sleep parameters and episodic memory consolidation. Sleep. 38:951–9. 25325488 10.5665/sleep.4748 PMC4434562

[bib3] Alonso A, McDorman SA, Romeo RR. (2024) How parent–child brain-to-brain synchrony can inform the study of child development. Child Dev Perspectives. 18:26–35. 10.1111/cdep.12494 PMC11486517 39421441

[bib4] Alqahtani F, Katsigiannis S, Ramzan N. (2020) Using wearable physiological sensors for affect-aware intelligent tutoring systems. IEEE Sensors J. 21:3366–78.

[bib5] Antle AN, Chesick L, Sridharan SK et al. (2018) East meets west: a mobile brain-computer system that helps children living in poverty learn to self-regulate. Pers Ubiquit Comput. 22:839–66.

[bib6] Antonenko PD, Niederhauser DS. (2010) The influence of leads on cognitive load and learning in a hypertext environment. Comput Hum Behav. 26:140–50.

[bib7] Arici F, Yildirim P, Caliklar Ş et al. (2019) Research trends in the use of augmented reality in science education: content and bibliometric mapping analysis. Comput Educ. 142:103647.

[bib8] Ayaz H, Shewokis PA, Curtin A et al. (2011) Using MazeSuite and functional near infrared spectroscopy to study learning in spatial navigation. J Vis Exp. 56:e3443. 10.3791/3443 PMC3227178 22005455

[bib9] Babiloni F, Astolfi L. (2014) Social neuroscience and hyperscanning techniques: past, present and future. Neurosci Biobehav Rev. 44:76–93. 22917915 10.1016/j.neubiorev.2012.07.006 PMC3522775

[bib10] Baceviciute S, Lucas G, Terkildsen T et al. (2022) Investigating the redundancy principle in immersive virtual reality environments: an eye-tracking and EEG study. J Comput Assist Lear. 38:120–36.

[bib11] Baddeley A . (2003) Working memory: looking back and looking forward. Nat Rev Neurosci. 4:829–39. 14523382 10.1038/nrn1201

[bib12] Balconi M, Angioletti L, Cassioli F. (2023) Hyperscanning EEG paradigm applied to remote vs. face-to-face learning in managerial contexts: which is better?. Brain Sci. 13:356. 36831899 10.3390/brainsci13020356 PMC9954592

[bib13] Berka C, Levendowski DJ, Cvetinovic MM et al. (2004) Real-time analysis of EEG indexes of alertness, cognition, and memory acquired with a wireless EEG headset. Int J Hum-Comput Interact. 17:151–70.

[bib14] Berka C, Levendowski DJ, Lumicao MN et al. (2007) EEG correlates of task engagement and mental workload in vigilance, learning, and memory tasks. Aviat Space Environ Med. 78:B231–44. 17547324

[bib15] Bevilacqua D, Davidesco I, Wan L et al. (2019) Brain-to-brain synchrony and learning outcomes vary by student–teacher dynamics: evidence from a real-world classroom electroencephalography study. J Cogn Neurosci. 31:401–11. 29708820 10.1162/jocn_a_01274

[bib16] Brockington G, Balardin JB, Zimeo Morais GA et al. (2018) From the laboratory to the classroom: the potential of functional near-infrared spectroscopy in educational neuroscience. Front Psychol. 9:1840. 30364351 10.3389/fpsyg.2018.01840 PMC6193429

[bib17] Brügger A, Richter KF, Fabrikant SI. (2019) How does navigation system behavior influence human behavior?. Cogn Res. 4:5. 10.1186/s41235-019-0156-5 PMC6374493 30758681

[bib18] Chen J, Qian P, Gao X et al. (2023) Inter-brain coupling reflects disciplinary differences in real-world classroom learning. Npj Sci Learn. 8:11. 37130852 10.1038/s41539-023-00162-1 PMC10154329

[bib19] Cheng B, Lin E, Wunderlich A et al. (2023) Using spontaneous eye blink-related brain activity to investigate cognitive load during mobile map-assisted navigation. Front Neurosci. 17:1024583. 36866330 10.3389/fnins.2023.1024583 PMC9971562

[bib20] Chuang HH, Liu HC. (2012) Effects of different multimedia presentations on viewers’ information-processing activities measured by eye-tracking technology. J Sci Educ Technol. 21:276–86.

[bib21] Cortina KS, Miller KF, Mckenzie R et al. (2015) Where low and high inference data converge: validation of CLASS assessment of mathematics instruction using mobile eye tracking with expert and novice teachers. Int J Sci Math Educ. 13:389–403.

[bib22] Coskun A, Cagiltay K. (2021) Investigation of classroom management skills by using eye-tracking technology. Educ Inf Technol. 26:2501–22.

[bib23] Dahlstrom‐Hakki I, Asbell‐Clarke J, Rowe E (2019) Showing is knowing: the potential and challenges of using neurocognitive measures of implicit learning in the classroom. Mind Brain Educ. 13:30–40.

[bib24] Davidesco I, Matuk C, Bevilacqua D et al. (2021) Neuroscience research in the classroom: portable brain technologies in education research. Educ Res. 50:649–56.

[bib25] Dikker S, Haegens S, Bevilacqua D et al. (2020) Morning brain: real-world neural evidence that high school class times matter. Soc Cogn Affect Neurosci. 15:1193–202. 33068110 10.1093/scan/nsaa142 PMC7745151

[bib26] Dikker S, Wan L, Davidesco I et al. (2017) Brain-to-brain synchrony tracks real-world dynamic group interactions in the classroom. Curr Biol. 27:1375–80. 28457867 10.1016/j.cub.2017.04.002

[bib27] Donthu N, Kumar S, Mukherjee D et al. (2021) How to conduct a bibliometric analysis: an overview and guidelines. J Business Res. 133:285–96.

[bib28] Feiler JB, Stabio ME. (2018) Three pillars of educational neuroscience from three decades of literature. Trends Neurosci Educ. 13:17–25.

[bib29] Feng X, Xu X, Meng Z et al. (2025) A rapid cortical learning process supporting students’ Knowledge construction during real classroom teaching. Adv Sci. 12, 2416610. 10.1002/advs.202416610 PMC12079370 39921272

[bib30] Filho E, Husselman T-A, Zugic L et al. (2022) Performance gains in an open skill video-game task: the role of neural efficiency and neural proficiency. Appl Psychophysiol Biofeedback. 47:239–51. 35688989 10.1007/s10484-022-09553-3

[bib31] Fischer KW, Goswami U, Geake J. (2010) The future of educational neuroscience. Mind Brain Educ. 4:68–80.

[bib32] Gao W, Wei T, Huang H et al. (2022) Toward a systematic survey on wearable computing for education applications. IEEE Internet Things J. 9:12901–15.

[bib33] Goldberg P, Schwerter J, Seidel T et al. (2021) How does learners’ behavior attract preservice teachers’ attention during teaching?. Teach Teach Educ. 97:103213.

[bib34] Guo J, Wan B, Wu H et al. (2022) A virtual reality and online learning immersion experience evaluation model based on SVM and wearable recordings. Electronics. 11:1429.

[bib35] Haar S, Faisal AA. (2020) Brain activity reveals multiple motor-learning mechanisms in a real-world task. Front Hum Neurosci. 14:354. 32982707 10.3389/fnhum.2020.00354 PMC7492608

[bib36] Haataja E, Garcia Moreno-Esteva E, Salonen V et al. (2019) Teacher's visual attention when scaffolding collaborative mathematical problem solving. Teach Teach Educ. 86:102877.

[bib37] Harley JM, Poitras EG, Jarrell A et al. (2016) Comparing virtual and location-based augmented reality mobile learning: emotions and learning outcomes. Education Tech Res Dev. 64:359–88.

[bib38] Hassan-Montero Y, De-Moya-Anegón F, Guerrero-Bote VP. (2022) SCImago Graphica: a new tool for exploring and visually communicating data. Profesional Inf/Inf Prof. 31:5.

[bib39] Holper L, Goldin AP, Shalóm DE et al. (2013) The teaching and the learning brain: a cortical hemodynamic marker of teacher–student interactions in the socratic dialog. Int J Educ Res. 59:1–10.

[bib40] Hood WW, Wilson CS. (2001) The literature of bibliometrics, scientometrics, and informetrics. Scientometrics. 52:291–314.

[bib41] Hou L, Pan Y, Zhu JJ. (2021) Impact of scientific, economic, geopolitical, and cultural factors on international research collaboration. J Informetrics. 15:101194.

[bib42] Janssen TWP, Grammer JK, Bleichner MG et al. (2021) Opportunities and limitations of mobile neuroimaging technologies in educational neuroscience. Mind Brain Educ. 15:354–70. 35875415 10.1111/mbe.12302 PMC9292610

[bib43] Jasińska KK, Guei S. (2018) Neuroimaging field methods using functional near infrared spectroscopy (NIRS) neuroimaging to study global child development: rural sub-Saharan Africa. J Vis Exp. 132.57165 10.3791/57165 PMC5912326 29443053

[bib44] Kandemir H, Kose H. (2022) Development of adaptive human–computer interaction games to evaluate attention. Robotica. 40:56–76.

[bib45] Kapaj A, Lanini-Maggi S, Hilton C et al. (2023) How does the design of landmarks on a mobile map influence wayfinding experts’ spatial learning during a real-world navigation task?. Cartogr Geographic Inform Sci. 50:197–213.

[bib46] Kaveh R, Schwendeman C, Pu L et al. (2024) Wireless ear EEG to monitor drowsiness. Nat Commun. 15:6520. 39095399 10.1038/s41467-024-48682-7 PMC11297174

[bib47] Khan A, Goodell JW, Hassan MK et al. (2022) A bibliometric review of finance bibliometric papers. Finance Res Lett. 47:102520.

[bib48] Klimesch W, Sauseng P, Hanslmayr S. (2007) EEG alpha oscillations: the inhibition–timing hypothesis. Brain Res Rev. 53:63–88. 16887192 10.1016/j.brainresrev.2006.06.003

[bib49] Krakauer JW, Hadjiosif AM, Xu J et al. (2019) Motor learning. Compr Physiol. 9:613–63. 30873583 10.1002/cphy.c170043

[bib50] Krigolson OE, Williams CC, Norton A et al. (2017) Choosing MUSE: validation of a low-cost, portable EEG system for ERP research. Front Neurosci. 11:109. 28344546 10.3389/fnins.2017.00109 PMC5344886

[bib51] Laal M, Laal M. (2012) Collaborative learning: what is it?. Procedia—Social Behavior Sci. 31:491–5.

[bib52] Lai M-L, Tsai M-J, Yang F-Y et al. (2013) A review of using eye-tracking technology in exploring learning from 2000 to 2012. Edu Res Rev. 10:90–115.

[bib53] Lau-Zhu A, Lau MP, McLoughlin G. (2019) Mobile EEG in research on neurodevelopmental disorders: opportunities and challenges. Dev Cogn Neurosci. 36:100635. 30877927 10.1016/j.dcn.2019.100635 PMC6534774

[bib54] Li J, Antonenko PD, Wang J. (2019) Trends and issues in multimedia learning research in 1996–2016: a bibliometric analysis. Edu Res Rev. 28:100282.

[bib55] Liu NH, Chiang CY, Chu HC. (2013) Recognizing the degree of human attention using EEG signals from mobile sensors. Sensors. 13:10273–86. 23939584 10.3390/s130810273 PMC3812603

[bib56] Liu X, Ardakani SP. (2022) A machine learning enabled affective E-learning system model. Educ Inf Technol. 27:9913–34. 10.1007/s10639-022-11010-x PMC8984673 35399782

[bib57] Lloyd-Fox S, Blasi A, Elwell CE. (2010) Illuminating the developing brain: the past, present and future of functional near infrared spectroscopy. Neurosci Biobehav Rev. 34:269–84. 19632270 10.1016/j.neubiorev.2009.07.008

[bib58] MacCoun RJ . (1998) Biases in the interpretation and use of research results. Annu Rev Psychol. 49:259–87. 15012470 10.1146/annurev.psych.49.1.259

[bib59] Mailhot T, Lavoie P, Maheu-Cadotte M-A et al. (2018) Using a wireless electroencephalography device to evaluate e-health and e-learning interventions. Nurs Res. 67:43–8. 29240659 10.1097/NNR.0000000000000260

[bib60] Maimon NB, Bez M, Drobot D et al. (2022) Continuous monitoring of mental load during virtual simulator training for laparoscopic surgery reflects laparoscopic dexterity: a comparative study using a novel wireless device. Front Neurosci. 15:694010. 35126032 10.3389/fnins.2021.694010 PMC8811150

[bib61] Matveeva N, Sterligov I, Lovakov A. (2022) International scientific collaboration of post-Soviet countries: a bibliometric analysis. Scientometrics. 127:1583–607.

[bib62] Mesmoudi S, Hommet S, Peschanski D. (2020) Eye-tracking and learning experience: gaze trajectories to better understand the behavior of memorial visitors. JEMR. 13:2. 10.16910/jemr.13.2.3 PMC7992043 33828795

[bib63] Nguyen JP, Shipley FB, Linder AN et al. (2016) Whole-brain calcium imaging with cellular resolution in freely behaving Caenorhabditis elegans. Proc Natl Acad Sci U S A. 113:E1074–81. 26712014 10.1073/pnas.1507110112 PMC4776509

[bib64] Page MJ, Mckenzie JE, Bossuyt PM et al. (2021) The PRISMA 2020 statement: an updated guideline for reporting systematic reviews. BMJ. 372:n71. 33782057 10.1136/bmj.n71 PMC8005924

[bib65] Pan Y, Novembre G, Olsson A (2022) The interpersonal neuroscience of social learning. Perspect Psychol Sci. 17:680–95. 34637374 10.1177/17456916211008429

[bib66] Pan Y, Novembre G, Song B et al. (2018) Interpersonal synchronization of inferior frontal cortices tracks social interactive learning of a song. Neuroimage. 183:280–90. 30086411 10.1016/j.neuroimage.2018.08.005

[bib67] Perpetuini D, Russo EF, Cardone D et al. (2022) Identification of functional cortical plasticity in children with cerebral palsy associated to robotic-assisted gait training: an fNIRS study. JCM. 11:6790. 36431267 10.3390/jcm11226790 PMC9692288

[bib68] Pessoa L . (2008) On the relationship between emotion and cognition. Nat Rev Neurosci. 9:148–58. 18209732 10.1038/nrn2317

[bib69] Pierno AC, Becchio C, Turella L et al. (2008) Observing social interactions: the effect of gaze. Social Neurosci. 3:51–9. 10.1080/17470910701563269 18633846

[bib70] Polat H, Topuz AC, Yıldız M et al. (2024) A bibliometric analysis of research on ChatGPT in education. IJTE. 7:59–85.

[bib71] Pouta M, Lehtinen E, Palonen T (2021) Student teachers’ and experienced teachers’ professional vision of students’ understanding of the rational number concept. Educ Psychol Rev. 33:109–28.

[bib72] Prieto LP, Sharma K, Kidzinski Ł et al. (2018) Multimodal teaching analytics: automated extraction of orchestration graphs from wearable sensor data. Computer Assisted Learning. 34:193–203. 10.1111/jcal.12232 PMC5909982 29686446

[bib73] Rayner K . (1998) Eye movements in reading and information processing: 20 years of research. Psychol Bull. 124:372. 9849112 10.1037/0033-2909.124.3.372

[bib74] Salminen-Saari JFA, Garcia Moreno-Esteva E, Haataja E et al. (2021) Phases of collaborative mathematical problem solving and joint attention: a case study utilizing mobile gaze tracking. ZDM Mathematics Edu. 53:771–84.

[bib75] Schneider B, Sharma K, Cuendet S et al. (2018) Leveraging mobile eye-trackers to capture joint visual attention in co-located collaborative learning groups. Int J Comput-Supp Collabor Learn. 13:241–61.

[bib76] Schöbel S, Saqr M, Janson A. (2021) Two decades of game concepts in digital learning environments–A bibliometric study and research agenda. Comput Edu. 173:104296.

[bib77] Scholkmann F, Kleiser S, Metz AJ et al. (2014) A review on continuous wave functional near-infrared spectroscopy and imaging instrumentation and methodology. Neuroimage. 85:6–27. 23684868 10.1016/j.neuroimage.2013.05.004

[bib78] Schroer SE, Yu C. (2023) Looking is not enough: multimodal attention supports the real-time learning of new words. Dev Sci. 26:e13290. 35617054 10.1111/desc.13290

[bib79] Seidel T, Stürmer K. (2014) Modeling and measuring the structure of professional vision in preservice teachers. Am Educ Res J. 51:739–71.

[bib80] Shamay-Tsoory SG, Mendelsohn A. (2019) Real-life neuroscience: an ecological approach to brain and behavior research. Perspect Psychol Sci. 14:841–59. 31408614 10.1177/1745691619856350

[bib81] Sharma K, Leftheriotis I, Giannakos M. (2020) Utilizing interactive surfaces to enhance learning, collaboration and engagement: insights from learners’ gaze and speech. Sensors. 20:1964. 32244457 10.3390/s20071964 PMC7180823

[bib82] Singh VK, Singh P, Karmakar M et al. (2021) The journal coverage of Web of Science, Scopus and dimensions: a comparative analysis. Scientometrics. 126:5113–42.

[bib83] Song Y, Chen X, Hao T et al. (2019) Exploring two decades of research on classroom dialogue by using bibliometric analysis. Comput Edu. 137:12–31.

[bib84] Stangl M, Maoz SL, Suthana N. (2023) Mobile cognition: imaging the human brain in the ‘real world.’. Nat Rev Neurosci. 24:347–62. 37046077 10.1038/s41583-023-00692-y PMC10642288

[doi102_196_032325] Sui J, Zhi D, Calhoun VD. (2023) Data-driven multimodal fusion: approaches and applications in psychiatric research. Psychoradiology, 3:kkad026. 10.1093/psyrad/kkad026. PMC10734907 38143530

[bib85] Sumardani D, Lin CH. (2023) Cognitive processes during virtual reality learning: a study of brain wave. Educ Inf Technol. 28:14877–96.

[bib86] Svoboda K, Li N. (2018) Neural mechanisms of movement planning: motor cortex and beyond. Curr Opin Neurobiol. 49:33–41. 29172091 10.1016/j.conb.2017.10.023

[bib87] Sweller J . (1988) Cognitive load during problem solving: effects on learning. Cogn Sci. 12:257–85.

[bib88] Takeuchi N, Mori T, Suzukamo Y et al. (2017) Integration of teaching processes and learning assessment in the prefrontal cortex during a video game teaching–learning task. Front Psychol. 7:2052. 28119650 10.3389/fpsyg.2016.02052 PMC5220187

[bib89] Tan SJ, Wong JN, Teo WP. (2023) Is neuroimaging ready for the classroom? A systematic review of hyperscanning studies in learning. Neuroimage. 281:120367. 37689175 10.1016/j.neuroimage.2023.120367

[bib90] Van Eck N, Waltman L. (2010) Software survey: vOSviewer, a computer program for bibliometric mapping. Scientometrics. 84:523–38. 20585380 10.1007/s11192-009-0146-3 PMC2883932

[bib91] Vigliocco G, Convertino L, De Felice S et al. (2024) Ecological brain: reframing the study of human behaviour and cognition. R Soc Open Sci. 11:240762. 39525361 10.1098/rsos.240762 PMC11544371

[bib92] Vošner HBž, Kokol P, Bobek S et al. (2016) A bibliometric retrospective of the Journal Computers in Human Behavior (1991-2015). Comput Hum Behav. 65:46–58.

[bib93] Wen D, Yuan J, Li J et al. (2023) Design and test of spatial cognitive training and evaluation system based on virtual reality head-mounted display with EEG recording. IEEE Trans Neural Syst Rehabil Eng. 31:2705–14. 37279134 10.1109/TNSRE.2023.3283328

[bib94] Whittier T, Willy RW, Sandri Heidner G et al. (2020) The cognitive demands of gait retraining in runners: an EEG study. J Mot Behav. 52:360–71. 31328698 10.1080/00222895.2019.1635983

[bib95] Xu J, Zhong B. (2018) Review on portable EEG technology in educational research. Comput Hum Behav. 81:340–9.

[bib96] Yan W, Zheng K, Weng L et al. (2020) Bibliometric evaluation of 2000–2019 publications on functional near-infrared spectroscopy. Neuroimage. 220:117121. 32619709 10.1016/j.neuroimage.2020.117121

[bib97] Yang LI, Huang J, Feng TI et al. (2019) Gesture interaction in virtual reality. Virtual Reality & Intelligent Hardware. 1:84–112.

[bib98] Yang X, Lin L, Cheng P-Y et al. (2018) Examining creativity through a virtual reality support system. Edu Tech Res Dev. 66:1231–54.

[bib99] Yoo G, Kim H, Hong S. (2023) Prediction of cognitive load from electroencephalography signals using long short-term memory network. Bioeng. 10:361. 10.3390/bioengineering10030361 PMC10044910 36978752

[bib100] Zhang J, Yu T, Wang M et al. (2023) Clinical applications of functional near-infrared spectroscopy in the past decade: a bibliometric study. Appl Spectrosc Rev. 59:908–34.

[bib101] Zhang Y, Hu Y, Ma F et al. (2024) Interpersonal educational neuroscience: a scoping review of the literature. Edu Res Rev. 42:100593.

